# Mind the Gap: Perceived Partner Responsiveness as a Bridge between General and Partner-Specific Attachment Security

**DOI:** 10.3390/ijerph17197178

**Published:** 2020-09-30

**Authors:** TeKisha M. Rice, Madoka Kumashiro, Ximena B. Arriaga

**Affiliations:** 1 Department of Human Development and Family Studies, University of Illinois at Urbana Champaign, Champaign, IL 61801, USA; 2 Department of Psychology, Goldsmiths, University of London, London SE14 6NW, UK; m.kumashiro@gold.ac.uk; 3 Department of Psychological Sciences, Purdue University, West Lafayette, IN 47907, USA; arriaga@purdue.edu

**Keywords:** perceived partner responsiveness, attachment security, attachment anxiety, attachment avoidance, romantic relationships

## Abstract

A core idea of attachment theory is that security develops when attachment figures are responsive to a person’s connection needs. Individuals may be more or less secure in different relationships. We hypothesized that individuals who perceive a current relationship partner as being responsive to their needs will feel more secure in that specific relationship, and that the benefits of perceived partner responsiveness would be more pronounced for individuals who generally feel insecure. The current study included 472 individuals (236 couples) in romantic relationships. Consistent with our predictions, individuals who perceived more responsiveness from their partner displayed lower partner-specific attachment anxiety and partner-specific avoidance, especially when they were generally insecure. These findings are discussed in terms of the conditions that promote secure attachment bonds.

## 1. Introduction

Adults whose experiences have led them to form secure attachment tendencies are generally trusting of others, comfortable with closeness, resilient when faced with adversity, at ease with themselves, and not afraid or overly pessimistic when pursuing personal goals [1]. Not everyone develops secure attachment tendencies. A core idea of attachment theory is that security develops when attachment figures are responsive to a person’s connection needs [2]. Recently, scholars have examined how individuals can become more secure, especially with romantic partners [3,4]. Romantic relationships provide an optimal context for enhancing attachment security when partners optimize meeting each other’s needs [5]. As suggested by models of effective caregiving [1,6], the current paper investigates whether perceiving a romantic partner as being responsive to one’s needs can strengthen attachment security with a current relationship partner, especially for individuals who may harbor insecurities about their close relationships in general. 

## 2. Overview of Attachment Theory

Attachment theory posits that individuals’ early experiences with caregivers shape future expectations of responsiveness from close others [2]. Bowlby [2] conceptualized these expectations as “working models,” which are elaborate schemas that encapsulate past attachment experiences. Individuals universally develop perceptions of themselves (their working model of self), of close others (relationship-specific models of others, such as relationship partners), and of others in general (global models of the others). Working models affect daily experiences by guiding a person’s perception of events they encounter, specific memories that are activated, and other cognitive, emotional, or behavioral responses [1]. Working models are especially influential when individuals feel distress or fear [2,7] and they underlie attachment “styles” of patterned responses that have been studied extensively because of their value in characterizing human bonds worldwide [8].

In adulthood, attachment styles are commonly conceptualized along dimensions of anxiety and avoidance [9]. When caregivers are consistently responsive to connection needs, individuals develop secure working models and exhibit low anxiety and low avoidance. As such, securely attached individuals tend to have positive representations of other people and are more confident in their ability to meet their partner’s needs [10]. Individuals who experience attachment security are more likely to seek intimacy from their partner [11], more comfortable with closeness [12], and more likely to engage in prosocial behavior [13].

However, when caregivers are inconsistent, inefficient, or inappropriate in their responsiveness to connection needs, individuals develop insecure working models. Individuals who chronically experience unreliable or inappropriate caregiving often develop attachment anxiety, characterized by chronic doubts about one’s self-worth and fear of not being loved or valued by others; these individuals yearn for closeness but fear abandonment [1,14]. Individuals who experience generally unresponsive or even hurtful caregiving often develop attachment avoidance, characterized by a general mistrust of others, emotional disengagement, and a desire to maintain independence [1,15].

Although the majority of the literature on adult attachment tends to portray individuals as being either chronically anxious or avoidant in their attachment orientations, attachment tendencies can change over time [16] and individuals can hold multiple different attachment representations [17,18,19,20]. People can develop different working models and corresponding attachment orientations about others in general, types of relationships such as friendships and romantic relationships in general, and specific others such as a current partner, friend, or parent. For example, a person could harbor beliefs that close others are generally unreliable (generally avoidant tendencies), yet feel that best friends can be counted upon (generally secure relational tendencies) and simultaneously worry about being abandoned by their current romantic partner (person-specific anxious tendencies).

Previous research suggests that the different attachment representations within an individual are somewhat related. Models of hierarchical structure suggest that general tendencies (working model toward others in general) may provide an overarching framework under which more specific relational tendencies (working models about different types of relationships) exist, followed by tendencies toward specific individuals [17,19]. For example, a person’s general global belief that other people will abandon them because they are not worthy (anxious tendencies) will also likely influence their belief that romantic partners are especially likely to abandon them. These beliefs may become self-fulfilling in specific relationships. For example, the person may engage in more clingy and conflictual behaviors when entering a romantic relationship that may increase the likelihood of the partner ending the relationship. Moreover, experiences within specific relationships may influence the general representation of close others. One study [18] revealed that attachment orientations toward specific individuals predicted 34% to 65% of the variance in general tendencies.

Previous research on multiple attachment representations is unclear on how an individual develops specific tendencies within a particular romantic relationship, especially if they tend to be generally insecure, as secure attachment tendencies tend to be more similar across different attachment representations within an individual, compared to insecure tendencies [21]. If general attachment tendencies do indeed provide an overarching framework for partner-specific attachment tendencies [17,19], what might explain how generally insecure individuals can become more secure in their specific relationships? We believe the key in explaining the “gap” lies with perceiving a partner as responsive.

## 3. Perceived Partner Responsiveness and Attachment Needs

Perceived partner responsiveness is a broad construct, defined as “a process by which individuals come to believe that relationship partners both attend to and react supportively to central, core defining features of the self” [22]. It is the feeling that partners understand, validate, and care about the most important aspects of the self. Perceived partner responsiveness is thought to lie at the heart of many important relationship processes as well as strengthen personal well-being and other important outcomes. Individuals who perceive their partners as responsive are more likely to experience positive affect [23], relational well-being [24], and long-term eudaimonic well-being (i.e., sense of meaning in life) [25].

Responsiveness is also thought to be vital to attachment security [22,26]. Research examining attachment in parent–child relationships suggests that parents’ responsive caregiving is a key predictor of children’s attachment [27,28]. As discussed above, responsive caregiving is thought to give rise to secure working models about the self and others, whereas inconsistent or unresponsive caregiving is thought to lead to insecure working models about the self and others [2,7]. In fact, scholars have argued that within parent–child relationships responsive caregiving may be the most influential variable in attachment and that a similar pattern may exist for adult close relationships [26]. Research on adult attachment has found that when individuals perceive that their partners respond supportively, they are more likely to experience feelings of security within their relationship [29].

Although inconsistent or unresponsive caregiving is thought to give rise to insecure working models of others and the self, dispositional attachment tendencies also influence perceptions of responsiveness [30,31]. Perceived partner responsiveness emphasizes “perception” of responsiveness, as many factors influence whether a partner’s behaviors are perceived as being sufficiently responsive to self’s needs at that particular instance [22]. Research on support processes finds that receiving support is not always beneficial as it can undermine self-esteem or self-efficacy [32], but perceiving support as being responsive had beneficial effects on both personal and relational outcomes [33]. In general, compared to acquaintances or strangers, individuals tend to expect more responsiveness from close relationship partners, who, for example, are expected to extend comfort in distressing times, remember and participate in significant events, and support personal goals [34,35]. In particular, insecurely attached individuals tend to perceive less responsiveness from their partners [30,31,36,37]. Perceiving such low levels of responsiveness is likely to maintain or increase feelings of insecurity.

In fact, recent theoretical models on changing attachment tendencies, like the Attachment Security Enhancement Model [3], suggest that partners can inadvertently play a role in maintaining attachment insecurity by reinforcing existing negative working models of the self or other. For example, when partners of avoidant individuals give in to preferences for less interdependence and intimacy, they may avoid immediate conflict. However, such acts are likely to leave the more secure partner feeling dissatisfied and result in poor relational quality or relationship dissolution. Such an outcome may reinforce the avoidant individual’s working model that others are unreliable.

The key to achieving attachment security lies in challenging existing working models. For example, longitudinal research findings suggest that anxious individuals become more secure over time when they perceive more encouragement of personal goals, which is contrary to their negative working model of the self as being incompetent [38]. Other research showed that avoidant expectant parents became more secure over time when they provided and received responsive support, presumably revising negative expectations of support and dependence [39]. 

Given that not perceiving consistent responsiveness is associated with developing both attachment anxiety and avoidance, we suggest that the key to achieving attachment security for both anxiously and avoidantly attached individuals is to perceive responsiveness from their partner. This task is often difficult, since even if their partners attempt to be responsive, insecurely attached individuals are less likely to perceive responsiveness from their partners [31,36,37]. Nevertheless, when insecurely attached individuals do perceive responsiveness from their partners, this will likely challenge existing working models that others are not responsive.

Such revisions to the working model are more likely to be made for the particular person who is perceived as being responsive, rather than necessarily be able to generalize to the working model of close others in general, although there may be some spill-over effects (e.g., become more secure toward romantic relationships in general). This is because previous research has found that tendencies toward specific individuals tend to predict behavior in those relationships better than assessments of general attachment tendencies [18,40,41].

Individuals who are generally secure in their romantic relationships are likely to feel secure in their specific relationships as well [19]. Thus, perceiving a specific romantic partner as responsive is particularly likely to revise working models of individuals who are typically generally insecure in their close relationships, and facilitate movement toward greater attachment security toward that specific partner. The current paper sought to examine the relationship between perceived partner responsiveness and greater attachment security, both at the levels of partner-specific attachment security and general attachment security (i.e., toward close others in general).

## 4. Current Study

Although responsiveness is thought to be central to the development of working models and subsequent attachment tendencies, surprisingly little empirical research has directly examined the impact of responsiveness on attachment tendencies. Previous research has also focused more on examining one type of attachment tendencies at a time (e.g., global, general relational role or partner-specific) rather than investigate what factors may account for the difference between general attachment tendencies and partner-specific tendencies. To our knowledge, previous empirical research has not examined how perceived partner responsiveness may help explain how people might become more secure in their specific relationships, especially for those who tend to be more generally insecure in their romantic relationships. To address this gap in the literature, we used cross-sectional data to examine two hypotheses:

**Hypotheses** **1** **(H1).** *Perceiving higher levels of partner responsiveness will predict greater general and partner-specific security (i.e., lower general and partner-specific anxiety and lower general and partner-specific avoidance).*

**Hypotheses** **2** **(H2).** *Perceived partner responsiveness will be positively associated with partner-specific attachment security for individuals who are generally less secure. That is, perceived partner responsiveness will be more strongly and negatively predictive of partner-specific attachment anxiety and avoidance among individuals who are generally more anxiously and avoidantly attached.*

Because behaviors intended to be responsive may not be perceived as such by recipients [22] we also sought to explore whether partner responsiveness would have the same effect on an individual’s partner-specific attachment anxiety and avoidance when reported by the partners themselves. We tested the above hypotheses by examining the effects of partner’s reports of their own responsiveness on the recipient’s partner-specific attachment security (i.e., attachment anxiety and avoidance), but we offer no a priori hypotheses.

## 5. Method

### 5.1. Participants

Participants included a convenience sample of 472 individuals (236 couples; 244 women, 228 men, 4 unidentified) obtained from three separate samples who participated in a larger study (*N* = 192 individuals, *N* = 134 individuals, *N* = 150 individuals). The study procedures and results of hypothesis tests were similar across the three samples, and therefore they were combined into one larger sample for the purposes of the current research.

Participants were recruited via flyers posted throughout the community and social media advertisements for the larger study that took place in a Midwestern US city. To be eligible, participants had to be at least 18 years old and in a monogamous relationship for at least 3 months where partners had regular contact with one another. On average, participants were approximately 25 years old (*SD* = 9.26). Participants predominately identified as White American (74.9%) and had been in their relationships for 54.70 months (*SD* = 73.65), which were mostly heterosexual (94%). Most participants had at least a high school education and were currently completing their bachelor’s degree (56.6%) or had their bachelor’s or an advanced degree (39%). Participants in the first study were significantly younger, less educated, and in their relationships for significantly less time than participants in latter two studies (Age: F (2, 467) = 6.18, *p* = 0.002; Education: F (2, 469) = 6.29, *p* = 0.002; Relationship Duration: F (2,447) = 24.18, *p* < 0.001. As stated above, the results of the hypothesis tests did not differ across the three samples). One of the samples differed from the other two on one of the four measures of security.

### 5.2. Procedures

Data were collected from 2016 to 2019. Participants attended a lab session in which they underwent informed consent procedures and then did a series of tasks for a larger study. The first task involved completing questionnaires that included variables analyzed in the current research. The questionnaires took approximately 30 min to complete. All procedures were approved by the Purdue University IRB (1505016116). Descriptive statistics for key variables are presented in Table 1.

### 5.3. Measures

*Partner-Specific Attachment*. Participants completed a 12-item version of the Experiences in Close Relationships Questionnaire (ECR) [42,43] with instructions and items pertaining to a current relationship partner. The partner-specific attachment anxiety subscale included six items (e.g., “I worry that my partner won’t care about me as much as I care about them”; α = 0.87), and the partner-specific attachment avoidance subscale also included six items (“I find it easy to depend on my partner”; α = 0.80). All items were rated on a 7-point scale from 1 (strongly disagree) to 7 (strongly agree).

*General Attachment*. Participants completed a similar version of the 12-item ECR questionnaire with instructions and items pertaining to “close relationships in general beyond your current partner.” The general attachment anxiety subscale included six items (α = α = 0.91), and the general attachment avoidance subscale included six items (0.88). All items were rated on a 7-point scale from 1 (strongly disagree) to 7 (strongly agree).

*Responsiveness*. Participants completed a 3-item measure rating their perception of their partner’s responsiveness [44] (α = 0.83): “My partner tries to make me feel valued as a person”, “My partner really tries to understand my concerns” and, “My partner listens to and comforts me”. Participants were asked to rate their own responsiveness toward their partner using 3 parallel items (e.g., “I try to make my partner feel valued as a person; α = 0.88). All items were rated on a 7-point scale from 1 (not at all) to 7 (very much so).

*Covariates.* In line with prior research examining attachment security, models testing predictors of one attachment dimension (e.g., attachment avoidance) controlled for the other attachment dimension (e.g., attachment anxiety). The models also controlled for each participant’s age, sex (male = 0, female = 1), and sample designation (Samples 1 and 3 = 0, sample 2 = 1).

### 5.4. Analytic Strategy

Descriptive and bivariate statistics were conducted for study variables in SPSS. Primary analyses were conducted in HLM 7 to account for the nested nature of the data (i.e., individuals comprising the observations), and individuals were nested within couples, which was modeled by including the couple number as a level-2 grouping variable [45,46]. A series of multiple regression models were tested, which included the main effects of independent variables and interaction terms. The independent variables were centered around the sample mean prior to creating interaction terms so that significant interactions could be interpreted in terms of specific levels compared to an average level. Significant interactions were decomposed by plotting simple effects at 1*SD* above and below the mean.

## 6. Results

### 6.1. Preliminary Analyses

Table 1 provides mean levels and standard deviation for all study variables. It also provides bivariate correlations among these variables. On average, general insecurity levels were higher than partner-specific insecurity levels: Partners reported more general attachment anxiety (*M* = 2.95, *SD* = 1.40) and general attachment avoidance (*M* = 3.44, *SD* = 1.29) than in their relationships with their current partners (attachment anxiety *M* = 1.84, *SD* = 1.00; attachment avoidance *M* = 1.95, *SD* = 0.83). Partners reported significantly less general attachment anxiety than general attachment avoidance, paired *t*(471) = −6.32, *p* < 0.001, 95% CI [−0.63, −0.33]. Similarly, participants reported significantly less partner-specific attachment anxiety than avoidance, paired *t*(467) = −2.22, *p* = 0.03, 95% CI [−0.21, −0.01]. Partner-specific attachment anxiety was significantly positively related to partner-specific attachment avoidance (*r* = 0.30) and general attachment anxiety (*r* = 0.48) but unrelated to general attachment avoidance. Partner-specific attachment avoidance was significantly and positively related to general attachment anxiety (*r* = 0.13) and general attachment avoidance (*r* = 0.38). Perceived partner responsiveness was positively correlated with partners’ reports of their own responsiveness (*r* = 0.33) and negatively related to every attachment dimension (see Table 1).

**Hypotheses** **1** **(H1).** *Perceived Partner Responsiveness Predicting Attachment Anxiety and Avoidance.*

We first examined the hypothesis that higher levels of perceived partner responsiveness would predict both greater general and partner-specific attachment security (i.e., lower general and partner-specific attachment anxiety and lower general and partner-specific attachment avoidance). We tested this with four models predicting each of the attachment variables (general versus specific attachment anxiety versus avoidance) while covarying out the other relevant attachment dimension.

As can be seen in Models 1 and 2 of Table 2, perceived partner responsiveness significantly predicted lower partner-specific attachment anxiety (*b* = −0.38, *p* = 0.04) and lower partner-specific attachment avoidance (*b* = −0.32, *p* = 0.04), while controlling for the other source of insecurity. Individuals who perceived their partners as being more responsive felt more secure (i.e., reported lower partner-specific attachment anxiety and avoidance) in their relationships. Perceived partner responsiveness significantly predicted lower general anxiety (*b* = −0.21, *p* = 0.02), but not general avoidance (*b* = −0.04, *p* = 0.70). Thus, H1 suggesting that perceived partner responsiveness predicts security (i.e., lower attachment anxiety and avoidance) was reliably supported for partner-specific security and had mixed results for general security.

**Hypotheses** **2** **(H2).** *Perceived Partner Responsiveness Will be Positively Associated with Partner-Specific Attachment Security for Individuals who are Generally Insecure.*

Next, we examined the hypothesis that higher levels of perceived partner responsiveness would predict greater partner-specific attachment security (i.e., lower partner-specific anxiety and avoidance) when individuals were generally more insecure. We tested this with two models predicting each of the partner-specific attachment variables (i.e., partner-specific attachment anxiety and partner-specific attachment avoidance) while covarying out the other relevant attachment dimension. Each model included perceived partner responsiveness as a predictor and the same covariates, but added the interaction between general attachment and perceived partner responsiveness.

**Figure 1:**
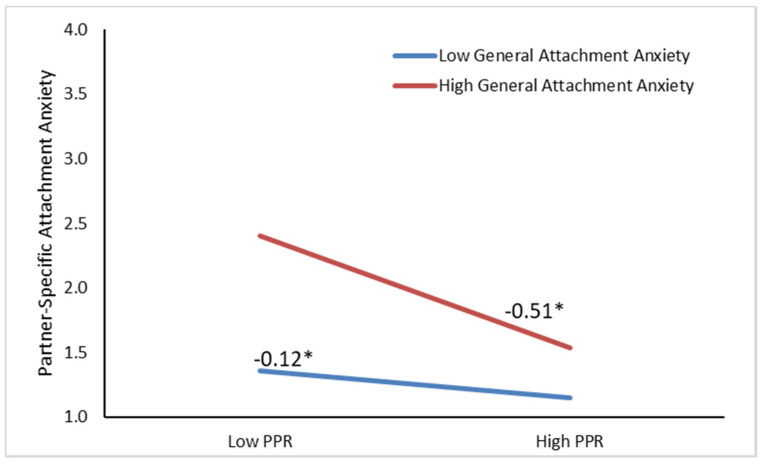
Predicting partner-specific attachment anxiety from perceived partner responsiveness as moderated by general attachment anxiety and controlling for partner-specific avoidance, age, sex, and study. PPR = Perceived Partner Responsiveness. * *p* < 0.05.

As can be seen in Model 1 of Table 3, perceived partner responsiveness significantly predicted lower partner-specific attachment anxiety (*b* = −0.31, *p* < 0.001). General attachment anxiety significantly predicted higher partner-specific attachment anxiety (*b* = 0.26, *p* < 0.001). The interaction between perceived partner responsiveness and general attachment anxiety was significant (*b* = −0.14, *p* < 0.001). Follow-up analyses to decompose the significant interaction indicated that among more generally anxiously attached individuals, perceived partner responsiveness was more strongly predictive of lower partner-specific attachment anxiety (see Figure 1), relative to individuals who were generally less anxiously attached. A follow-up model testing moderation by sex indicated that this was more pronounced for women than for men, but the association of perceived partner responsiveness remained significant for both men and women and therefore the model without significant moderation by sex was retained.

**Figure 2:**
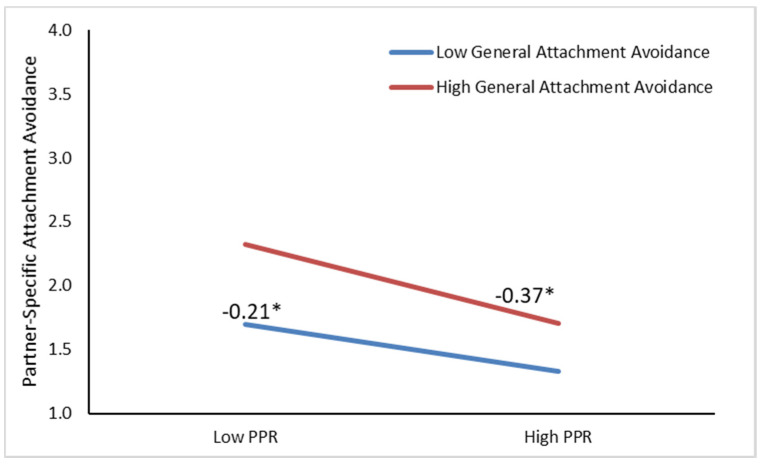
Predicting partner-specific attachment avoidance from perceived partner responsiveness as moderated by general attachment anxiety and controlling for partner-specific avoidance, age, sex, and study. PPR = Perceived Partner Responsiveness. * *p* < 0.05.

Model 2 of Table 3 shows that perceived partner responsiveness significantly predicted lower partner-specific attachment avoidance (*b* = −0.29, *p* < 0.001). General attachment avoidance significantly predicted higher partner-specific attachment avoidance (*b* = 0.19, *p* < 0.001). The interaction between perceived partner responsiveness and general attachment avoidance was significant (*b* = −0.06, *p* = 0.05). Follow-up analyses to decompose the significant interaction indicated that among more generally avoidantly attached individuals, perceived partner responsiveness was more strongly predictive of lower partner-specific attachment avoidance (see Figure 2), relative to individuals who were generally less avoidantly attached. A follow-up model testing moderation by sex indicated that this did not differ as a function of the participant’s sex. Thus, H2 suggesting that perceived partner responsiveness predicts greater partner-specific security (i.e., lower attachment anxiety and avoidance) when individuals are generally less secure was reliably supported (see Table 3).

### 6.2. Exploratory Analyses

Exploratory analyses also predicted a person’s own level of partner-specific attachment anxiety and avoidance as in the models above, but utilized the partner’s report of their responsive behavior rather than the person’s (recipient’s) perception of the partner’s responsiveness. These models thus tested cross-partner effects to determine whether what one person reports doing in terms of responsiveness predicts the other person’s security.

Two initial models were run, one predicting partner-specific attachment anxiety and the second predicting general attachment anxiety. Each model included the partner’s report of their own responsive behavior toward the recipient as a predictor and the same covariates as the analyses above. Partners’ reports of their own responsiveness toward recipients significantly predicted less partner-specific attachment anxiety among recipients (*b* = −0.41, *p* < 0.001) and less general attachment anxiety among recipients (*b* = −0.32, *p* = 0.002). An additional model tested whether the association of the partner’s responsiveness (as reported by the partner) was more pronounced when the recipient was generally anxiously attached (i.e., the same model predicting partner-specific anxiety, adding a general attachment anxiety main effect and general attachment anxiety by partner responsiveness interaction term). The interaction was not significant (*b* = 0.09, *p* = 0.16).

Two initial models were run predicting partner-specific attachment avoidance and general attachment avoidance. Partners’ reports of their own responsiveness toward recipients did not predict a recipient’s attachment avoidance toward that partner (*b* = −0.09, *p* = 0.15) nor the recipient’s general attachment avoidance (*b* = 0.09, *p* = 0.29). These effects were in the predicted direction but not significant. Thus, individuals whose partners reported being more responsive had significantly lower levels of partner-specific and general attachment anxiety, but the partner’s responsive behavior did not affect a recipient’s level of attachment avoidance. The detailed results of these analyses are available from the first author.

## 7. Discussion

The present study builds on prior theoretical work by empirically examining perceived partner responsiveness as an enhancer of attachment security. Our findings suggest that: (a) perceived partner responsiveness is consistently predictive of enhanced partner-specific attachment security and lower general attachment anxiety; (b) perceiving a partner as responsive offers more benefits for individuals who are generally insecure; and (c) across partners, one partner’s report of their own highly responsive behavior predicts lower levels of the other person’s partner-specific attachment anxiety.

First, we found strong support for one part of our first hypothesis that perceived partner responsiveness would predict greater partner-specific security and found mixed support in predicting general attachment security toward close others. People who perceived their partners as more responsive reported lower levels of both partner-specific attachment anxiety and avoidance, but only lower levels of general attachment anxiety. These findings are important as attachment theorists, starting with Bowlby [2,7], having placed responsive caregiving and support as being at the heart of attachment theory and critical to forming and maintaining secure working models of the self and others [24,31]. Such working models then contribute to developing more stable, chronic attachment tendencies, especially toward specific partners.

Most research on adult attachment examines social support processes or responsive support toward goal attainment [4] but has not examined the general perception of partner responsiveness toward the self (e.g., feeling understood, validated, and cared for). The current findings show that attachment security may be bolstered outside of specific support or caregiving contexts. Specifically, perceiving responsiveness from a partner in general contexts is associated with greater partner-specific attachment security and may lower general levels of attachment anxiety toward close others. These findings are also in line with prior literature showing higher associations between various relationship processes within a particular relationship and person-specific attachment security than with general attachment security [18,19].

Why might specific experiences with a partner also affect general attachment anxiety but not avoidance? This may be because anxiously attached individuals have somewhat of a more optimistic working model toward close others in comparison to avoidantly attached individuals, where they hope that others will offer support and love, despite believing that they themselves are not worthy of such care (i.e., negative working model of the self) [1]. Thus, anxiously attached individuals may have more malleable working models, allowing them to believe that if a current partner can offer the kind of responsiveness they crave, others may be able to do so as well. Indeed, a previous study examining multiple attachment representations showed that attachment anxiety (or working model of the self-dimension) was more closely related among different types of relationships than was the case for attachment avoidance [21].

On the contrary, avoidantly attached individuals have a negative working model of others where they view people as undependable. Individuals who are avoidantly attached often inflate their working model of self, viewing themselves as self-reliant in an effort to protect themselves from feeling rejected by others [1]. Thus, avoidantly attached individuals may be more likely to compartmentalize their experiences with a specific partner and less likely to expect similar interactions with close others in general. Instead, individuals who are avoidantly attached may need to have similarly responsive experiences with multiple close relationship partners in order for their working models of close others to also be revised. Interestingly, findings of a longitudinal study suggest that trust was associated with lower levels of attachment avoidance toward general romantic relationships a year later [38]. Since our study examined general attachment avoidance toward close others, it may be the case that perceived partner responsiveness from a specific partner may similarly be able to influence working models toward romantic relationships in general, but not necessarily to all close relationships.

This differential pattern between attachment anxiety versus avoidance also extends to partner reports of their own responsiveness. For anxiously attached individuals, responsiveness may not just be in the eye of the beholder (i.e., based only on their own perceptions). Partner reports of their own responsiveness predicted significantly lower attachment anxiety in the other person (both general and partner-specific anxiety). This was not the case for attachment avoidance.

Partner reports may influence an individual’s attachment anxiety more than attachment avoidance, because partners of anxious individuals may need to try harder to show responsiveness in order to meet their partners’ needs. On the other hand, partners of avoidantly attached individuals may somewhat cease efforts at being responsive if it seems their efforts are not being appreciated or rejected. As anxiously attached individuals are more likely to have a negative working model of the self, where they are constantly in fear of being rejected by close partners, they also crave and demand constant reassurance of love and commitment [1,14]. Consequently, anxiously attached individuals are more likely to be more vocal about their needs than avoidant individuals, who are more likely to reject intimacy, shun support processes, and less likely to self-disclose [1,15]. Thus, partners of anxiously attached individuals may better understand their anxious partner’s needs, compared to partners of avoidantly attached individuals who may not be aware of the avoidant individual’s central, core inner thoughts and needs. Such contrasting responses of partners of anxiously or avoidantly attached individuals may then directly influence the person’s attachment security levels.

Second, we tested and found support for our novel hypothesis that perceiving partner responsiveness will be especially likely to offer benefits in achieving greater partner-specific security (for both attachment anxiety and avoidance) for individuals who still reported more general insecurity. This finding is especially notable given that individuals who exhibit insecure tendencies are less likely to perceive their partners as caring and responsive [37]. Yet, our findings suggest that when insecure individuals do perceive responsiveness from their partner, they are more likely to feel secure in their relationships.

It should be noted that we predicted this novel hypothesis because insecurely attached individuals are in need of greater revisions to their working models toward security compared to those who are already relatively secure. Partner responsiveness provides new experiences that contradict insecure working models and therefore may cause those models to change [38,39]. Generally, securely attached individuals are already more likely to report greater security across different types of relationships [21], and they are already more likely to perceive responsiveness compared to their insecure counterparts [31,36]. Thus, it is critical that generally insecure individuals perceive responsiveness from their partner if they are to experience greater security. Otherwise, insecure individuals may report lower levels of responsiveness from their partners and remain unlikely to revise their negative working models about the self and others.

Perceptions of responsiveness seem to be key, as partner reports of their own responsiveness did not appear to benefit individuals who are more generally insecure in achieving greater partner-specific attachment insecurity. Our findings thus suggest that although partner reports of their own responsiveness were associated with lower levels of both general and partner-specific attachment anxiety, individuals who were generally more insecure showed enhanced partner-specific attachment insecurity only when they perceived responsiveness from their partner.

The current findings are particularly noteworthy, as prior research has indicated that the key to long-term improvements in attachment security may differ for anxiously and avoidantly attached individuals [38]. Our findings suggest that perceived partner responsiveness may help revise insecurities in the working model of self and the working model of others. Of course, the findings of the current study do not reveal if perceived partner responsiveness revises working models of both the self and others in a similar manner for attachment anxiety and avoidance. Individuals who are anxiously attached have insecure working models of the self, which leaves them fearing that they may not be valued by others and craving more closeness than others can provide [14], whereas individuals who are avoidantly attached have negative working models of others, which leads them to shun intimacy and dependence [1]. Perceiving responsiveness from partners may help anxiously attached individuals feel more assured of their self-value and help avoidantly attached individuals feel that others are dependable.

Nevertheless, previous longitudinal findings suggest both trust and goal validation mattered for both attachment anxiety and avoidance but with different short-term and long-term effects. When assessed concurrently, trust was more strongly associated with less anxious attachment and perceiving one’s personal goals as validated by a partner was more strongly associated with less attachment avoidance, but the effects were reversed a year later [38]. Given that perceived partner responsiveness lies at the heart of many important relationship processes [22], it may be the case that perceived partner responsiveness may influence other closely related relationship constructs (e.g., trust, responsive goal support) that may help bolster attachment security, and this may differ for attachment anxiety and avoidance at different points in the relationship.

Overall, the findings of this study show the need to empirically examine basic processes, such as perceived partner responsiveness, that have been theorized to be at the core of many relationship processes, including theories of attachment [22]. Since this was a correlational study, it remains to be seen if over time, regularly perceiving partners as responsive may not only boost partner-specific attachment security, but may also enhance general security or security toward types of relationships.

### Limitations and Future Directions

The findings of this study are important since this is one of the first empirical studies to address how perceived partner responsiveness may help explain the “gap” between different attachment representations for general and partner-specific attachment security. Despite the importance of these findings, this study has some limitations. First, this study was cross-sectional and cannot speak to perceived partner responsiveness as a causal predictor of attachment security. Longitudinal and experimental investigations are necessary to isolate the causal influence of perceived partner responsiveness and examine how it operates with other relational processes at different points in relationships to bolster attachment security. In particular, deeply entrenched attachment styles are resistant to change, requiring repeated experiences that contradict insecure working models [3]. As such, most studies require observing an extended period of time to reveal changes [38] and may hone in on important life transition periods such as parenthood [39,47]. Given that such studies have still been correlational in nature, an ideal future study would involve a long-term clinical randomized trial of couples who have been randomly assigned to receive some kind of relationship-enhancing or otherwise working model revising treatment and followed up over time.

Second, the current findings only examined partner-specific and general attachment tendencies but did not assess other types of relationships or attachment tendencies toward romantic partners in general. Perceived partner responsiveness from a specific partner may play a larger role in influencing working models toward that particular type of relationships (i.e., romantic relationships in general), in a similar way that trust reduced attachment security toward romantic partners in general [38]. This may be particularly the case for romantic relationships, as a person typically has only one romantic partner at a time, whereas people are more likely to have multiple different types of friendships that may make it more difficult for person-specific experiences to spill over into revising working models toward friendships. Future research should examine the role of perceived partner responsiveness in minimizing the gap between person-specific and relational role attachment tendencies across different types of relationships as well as and compare partner-specific, relational role, and general global attachment representations.

Third, we measured perceived partner responsiveness globally and abstractly, and did not assess what behaviors may be perceived as being responsive. Perceiving responsiveness can vary by context or relationship partners [22], such that the same act, such as providing flowers, may be perceived as being very unresponsive or responsive, depending on who is giving the flowers and why. Given that insecure individuals have already been shown to perceive less responsiveness compared to their secure counterparts [30,31,36], future research should examine more specifically the type of partner behaviors insecure individuals perceive as responsive, and whether this depends on the type of attachment insecurity. Anxiously attached individuals may need to regularly perceive responsiveness that communicates value, validation, and care whereas avoidantly attached individuals may need to regularly perceive responsiveness that communicates trust and reinforces the value of emotional closeness [3].

Similarly, the current study examined only the partner’s report of their own responsiveness as a somewhat objective measure of responsiveness. Compared to partners of secure individuals who may perceive simple kind acts or words as being responsive, partners of insecure individuals are likely to also notice that their attempts at being responsive are not being perceived as such and may consequently report lower levels of responsiveness provision. Future research should consider observer reports and other more objective measures of responsiveness and what behaviors contribute to perceiving responsiveness.

Finally, our findings may not be generalized to diverse groups of individuals, relationship types, or cultural contexts. The sample overrepresented individuals who identified as being of European-American descent, heterosexual, young, and college educated, and who resided in the midwestern part of the USA. Future studies should examine whether such variations in social context produce similar or differing findings.

## 8. Conclusions

The current study is one of the first to show empirical support for the assumption that perceived partner responsiveness plays a central role in strengthening attachment security. These findings have important implications for future studies and interventions aimed at addressing attachment insecurity, especially among individuals in romantic relationships. Practitioners might emphasize the importance of responsiveness to partners of insecure individuals and work with insecure individuals on how they can reinterpret partner behaviors as being responsive to their needs, much like how exercises that helped reframe partner compliments fostered relationship security for low self-esteem individuals who typically resist positive feedback from their partners [48].

Attachment insecurities may be resistant to change but the findings of this study contribute to a growing body of literature concerned with the ways that individuals can become more secure in their relationships, and perhaps become more secure in general. Our findings also contribute to the growing literature emphasizing the centrality of perceived partner responsiveness for close relationship processes [22,49]. Given that both attachment security and perceived partner responsiveness are associated with positive relational and personal outcomes [1], the findings of this study may help contribute to the growing interest in learning more about the underlying processes that contribute to overall well-being.

## Figures and Tables

**Table 1:** Means, standard deviations, and bivariate intercorrelations for study variables.

Variable	Mean (*SD*)	1	2	3	4	5
Partner-specific attachment anxiety	1.84 (1.00)	--				
2. Partner-specific attachment avoidance	1.95 (0.83)	0.30 *	--			
3. General attachment anxiety	2.95 (1.40)	0.46 *	0.13 *	--		
4. General attachment avoidance	3.44 (1.29)	0.07	0.38 *	0.25 *	--	
5. Perceived partner responsiveness	6.33 (0.85)	−0.18 *	−0.34 *	−0.10 *	−0.14 *	--
6. Own responsiveness reported by partner	6.46 (0.63)	−0.29 *	−0.15 *	−0.13 *	−0.01	0.33 *

Note: * *p* < 0.05. All variables ranged from 1 to 7. *SD* = standard deviation.

**Table 2:** Predicting partner-specific and general attachment anxiety and avoidance from perceived partner responsiveness.

	Model 1:			Model 2:			Model 3:			Model 4:		
	Partner-Specific			Partner-Specific			General			General		
	Attachment Anxiety			Attachment Avoidance			Attachment Anxiety			Attachment Avoidance		
	Coefficient (*SE*)		*t*	Coefficient (*SE*)		*t*	Coefficient (*SE*)		*t*	Coefficient (*SE*)		*t*
Intercept	4.01 *	(0.59)	6.81	3.85 *	(0.47)	8.19	3.37 *	(0.63)	5.37	3.10 *	(0.60)	5.16
Dyad	0.00	(0.00)	−1.05	0.00	(0.00)	−0.61	0.00	(0.00)	−0.18	0.00	(0.00)	−0.87
Age	−0.01 *	(0.00)	−2.28	0.00	(0.00)	0.51	−0.02 *	(0.01)	−2.79	0.01	(0.01)	1.36
^a^ Sex	0.23 *	(0.08)	2.94	−0.30 *	(0.07)	−4.26	0.68 *	(0.13)	5.41	−0.72 *	(0.11)	−6.32
^b^ Sample	−0.22 *	(0.08)	−2.79	−0.09	(0.08)	−1.10	0.00	(0.13)	0.03	−0.04	(0.12)	−0.38
Partner-specific anxiety	-	-	-	0.15	(0.04)	3.26	-	-	-	-	-	-
Partner-specific avoidance	0.20	(0.06)	3.17	-	-	-	-	-	-	-	-	-
General anxiety	-	-	-	-	-	-	-	-	-	0.26 *	(0.05)	5.18
General avoidance	-	-	-	-	-	-	−0.30 *	(0.05)	6.00	-	-	
PPR	−0.38 *	(0.08)	−4.65	−0.32 *	(0.06)	−5.09	−0.21 *	(0.09)	−2.42	−0.04	(0.08)	−0.44

Note: * *p* < 0.05. Coefficients are unstandardized, and SE = standard error. PPR = Perceived Partner Responsiveness; dyad refers to a random number assigned to both members of a couple; ^a^ 0 = male and 1 = female; ^b^ 0 = Samples 1 and 3 = 0, Sample 2 = 1. Models predicting attachment anxiety (partner-specific or general) covaried out attachment avoidance (and vice versa in predicting attachment avoidance and covarying attachment anxiety) in order to control for each source of insecurity and allow for inferences about greater security.

**Table 3:** Partner-specific attachment anxiety and avoidance predicted by perceived partner responsiveness for generally anxiously attached and generally avoidantly attached individuals.

	Model 1:			Model 2:		
	Partner-Specific			Partner-Specific		
	Attachment Anxiety			Attachment Avoidance		
	Coefficient (*SE*)		*t*	Coefficient (*SE*)		*t*
Intercept	1.61 *	(0.15)	10.81	1.76 *	(0.14)	12.49
Dyad	0.00	(0.00)	−0.92	0.00	(0.00)	−0.18
Age	−0.01	(0.00)	−1.66	0.00	(0.00)	0.37
^a^ Sex	0.09	(0.07)	1.22	−0.19 *	(0.07)	−2.89
^b^ Sample	−0.21 *	(0.07)	−2.94	−0.08	(0.07)	−1.06
Partner-specific anxiety	-	-	-	0.14 *	(0.04)	3.30
Partner-specific avoidance	0.19 *	(0.06)	3.19	-	-	-
PPR	−0.31 *	(0.05)	−6.22	−0.29 *	(0.06)	−5.17
General anxiety	0.26 *	(0.03)	8.15	-	-	-
General anxiety × PPR	−0.14 *	(0.03)	−5.35	-	-	-
General avoidance	-	-	-	0.19 *	(0.03)	6.89
General avoidance × PPR	-	-	-	−0.06 *	(0.03)	−1.96

Note: * *p* < 0.05. Coefficients are unstandardized, and *SE* = standard error. PPR = Perceived Partner Responsiveness; dyad refers to a random number assigned to both members of a couple; ^a^ 0 = male and 1 = female; ^b^ 0 = Samples 1 and 3 = 0, Sample 2 = 1. Models predicting partner-specific attachment anxiety covaried out attachment avoidance, and vice versa when predicting attachment avoidance and covarying attachment anxiety, in order to control for each source of insecurity and allow for inferences about greater security.
